# Prevalence of β-lactamase-encoding genes and molecular typing of *Acinetobacter baumannii* isolates carrying carbapenemase OXA-24 in children

**DOI:** 10.1186/s12941-021-00480-5

**Published:** 2021-10-26

**Authors:** Neda Yousefi Nojookambari, Mehrzad Sadredinamin, Razieh Dehbanipour, Zohreh Ghalavand, Gita Eslami, Maryam Vaezjalali, Bahram Nikmanesh, Sajjad Yazdansetad

**Affiliations:** 1 Department of Microbiology, School of Medicine, Shahid Beheshti University of Medical Sciences, Tehran, Iran; 2 Department of Medical Laboratory Sciences, School of Allied Medical Sciences, Tehran University of Medical Sciences, Tehran, Iran; 3 Laboratory Sciences Research Center, Golestan University of Medical Sciences, Gorgan, Iran

**Keywords:** Antimicrobial susceptibility, Pediatrics, *Acinetobacter baumannii*, RAPD-PCR, β-Lactamases, *bla*_OXA-24-like_

## Abstract

**Background:**

β-Lactam antibiotics have been broadly used for the treatment of *Acinetobacter baumannii* infections, resulting in development of β-lactam inactivating β-lactamases. Here, we described antibiotic resistance rate, prevalence of β-lactamase-encoding genes, and clonal relationships of *A. baumannii* strains isolated from children referred to Children’s Medical Center in Tehran, Iran, during 2019–2020.

**Methods:**

A total of 60 non-replicate *A. baumannii* isolates were recovered from clinical specimens of pediatric patients. Antibiotic susceptibility testing was done by the disc diffusion method. Colistin susceptibility of isolates was performed by the broth microdilution method. β-lactamase-encoding genes were characterized by PCR. The presence of IS*Aba1* element upstream of the several oxacillinase genes was also checked. Genetic relatedness of isolates was determined by using random amplification of polymorphic DNA (RAPD) typing.

**Results:**

The antimicrobial susceptibility tests showed that 83.3% of *A. baumannii* isolates were MDR, and 40% XDR. Both MDR and XDR *A. baumannii* isolates were susceptible to colistin. The frequency of *bla*_OXA-51-like_, *bla*_OXA-23-like_, *bla*_TEM_, *bla*_OXA-24-like_, *bla*_PER_, *bla*_SHV_, *bla*_CTX-M_, *bla*_OXA-58-like_, and *bla*_IMP_ was 100, 93.33, 60, 36.67, 28.33, 8.33, 5, 3.33, and 1.67%, respectively. Coexistence of IS*Aba1*/*bla*_OXA-23-like_ and IS*Aba1*/*bla*_OXA-51-like_ was observed in 65% and 85% of isolates, respectively. RAPD analysis revealed 4 common types and 2 single types of *A. baumannii* isolates.

**Conclusions:**

The multiple clones harboring *bla*_OXA-23-like_, IS*Aba1*-*bla*_OXA-51-like_, and IS*Aba1*-*bla*_OXA-23-like_ were responsible for the spread of *A. baumannii* isolates in our clinical wards. Dissemination of the well-established clones is worrisome and would become therapeutic challenges due to the possible transferring genetic elements associated with resistance.

**Supplementary Information:**

The online version contains supplementary material available at 10.1186/s12941-021-00480-5.

## Background

*Acinetobacter baumannii*, a known serious human pathogen, is the most important for rising rates of hospital-acquired infections and rapid development of antibiotic and antimicrobial resistance worldwide [1]. *A. baumannii* is usually transmitted by medical staff-to-patient contact and exposure to contaminated surfaces; nevertheless, aerosol route from colonized patients has also been reported [2]. The ability of *A. baumannii* for environmental persistence and desiccation resistance makes it a successful pathogen to spread in healthcare settings [3]. Healthcare-associated pneumonia and bacteremia are the most common clinical manifestations of *A. baumannii* infections; however, other infection types, including wound infections, urinary tract infections, endocarditis, meningitis, endophthalmitis, and osteomyelitis have also been seen in adults and children [4].

Recently, multidrug-resistant (MDR) *A. baumannii* has emerged as a significant pathogen in children with high morbidity and mortality and caused serious therapeutic problems due to the rapid acquisition of a large diversity of antibiotic-resistance genes [5]. β-lactam antibiotics (e.g., penicillins, cephalosporins, carbapenems, and monobactams) are empirically prescribed for the treatment of infections caused by *A. baumannii*. However, several β-lactam hydrolyzing groups such as class A β-lactamases, ESBLs, (e.g., TEM, SHV, CTX-M, PER, VEB, and GES), class B β-lactamases, MBLs, (e.g., IMP, VIM, GIM, and NDM), class C AmpC cephalosporinases, and class D carbapenemases/oxacillinases, OXA types, (e.g., OXA-23-like, OXA-24-like, OXA-51-like, and OXA-58-like) were most frequently described in *A baumannii *[6, 7]. Carbapenems possess potent antimicrobial activity against MDR *A. baumannii*, although, new carbapenem-hydrolyzing beta-lactamases have arisen with increasing carbapenem use resulted in a higher risk of treatment failure [8].

Insertion sequences (ISs) cause mutations and genome rearrangements resulting in spread of resistance and enhancing virulence determinants in *Acinetobacter* species. Previous studies have been showing that IS*Aba1* element is responsible for the transfer and overexpression of carbapenem resistance genes (e.g., *bla*_OXA-51-like_, *bla*_OXA-23-like_, and *bla*_OXA-58-like_) in *A. baumannii *[9, 10].

The rising rate of drug-resistant *A. baumannii* in pediatric patients is alarming and limiting the treatment options. The aim of this study was to determine in vitro activity of antibiotics, presence of β-lactamase-encoding genes, and genetic diversity of *A. baumannii* strains isolated from children referred to Children’s Medical Center in Tehran, Iran, during 2019–2020.

## Materials and methods

### Clinical specimens, bacterial isolates, and identification

This study was conducted on 60 non-duplicate *A. baumannii* isolates obtained from pediatric patients, aged from 4 days–14 years old, and hospitalized in different wards of Children’s Medical Center in Tehran, during April 2019 until December 2020. The isolates recovered from various clinical specimens were identified by the routine microbiological tests as *A. baumannii*. Then isolates were confirmed by PCR amplification of partial RNA polymerase β-subunit (*rpoB*) gene using a set of forward and reverse primers of 5′-TAYCGYAAAGAYTTGAAAGAAG-3′ and 5′-CMACACCYTTGTTMCCRTGA-3′, as described previously [11]. All identified *A. baumannii* strains were stored at − 70 °C until further investigations.

### Antimicrobial susceptibility test

Antimicrobial susceptibility testing was carried out by the Kirby–Bauer disc diffusion method according to Clinical and Laboratory Standards Institute (CLSI) [12] and breakpoint interpretations for including amikacin (30 µg), gentamicin (10 µg), tobramycin (10 µg), cotrimoxazole (trimethoprim–sulfamethoxazole) (25 µg), tigecycline (15 µg), doxycycline (30 µg), tetracycline (30 µg), minocycline (30 µg) ceftriaxone (30 µg), ceftazidime (30 µg), cefepime (30 µg), cefotaxime (30 µg), piperacillin/tazobactam (100/10 µg), ampicillin-sulbactam (10/10 µg), imipenem (10 µg), meropenem (10 µg), ciprofloxacin (5 µg), levofloxacin (5 µg), (Mast Group Ltd., Bootle, UK). The minimum inhibitory concentrations (MICs) of colistin (Colistin sulfate salt powder, Sigma-Aldrich, St. Louis, MO, USA) were determined using the broth microdilution method according to CLSI guidelines (CLSI, 2020). *Escherichia coli* ATCC 25922 and *Pseudomonas aeruginosa* ATCC 27,853 strains were used as the standard strains.

### Detection of β-lactamase-encoding genes

Genomic DNA was extracted by BioFact™ Genomic DNA Prep Kit GD141-100 (BioFact, Daejeon, Korea) according to the manufacturer’s recommendations. The primer sequences, product sizes, and annealing temperatures for *bla*_TEM_, *bla*_SHV_, *bla*_CTX-M_, *bla*_VEB_, *bla*_PER_, *bla*_GES_, *bla*_IMP_, *bla*_VIM_, *bla*_NDM_, IS*Aba1*, *bla*_OXA-51-like_, *bla*_OXA-24-like_, *bla*_OXA-23-like_, *bla*_OXA-58-like_, IS*Aba1*/*bla*_OXA-23-like_, and IS*Aba1*/*bla*_OXA-51-like_ gene fragments are presented in Table 1. The presence of IS*Aba1* insertion upstream of *bla*_OXA-51-like_ and *bla*_OXA-23-like_ genes was explored by using the forward primer for IS*Aba1* and the reverse primers for *bla*_OXA-51-like_ and *bla*_OXA-23-like_. PCR was carried out using the ready-to-use 2X *Taq* DNA Polymerase Master Mix RED (Ampliqon, Denmark) in a 25 mL total volume reaction containing ~ 30 ng of DNA template and 10 pmol/mL of forward and reverse primers. PCR was performed under conditions of initial denaturation at 94 °C for 5 min, 35 cycles of denaturation at 94 °C for 45 s, annealing ranging from 47 to 58 °C for 45 s, extension at 72 °C for 45 s, and final extension at 72 °C for 5 min. PCR products were separated on agarose gel 1.5% by electrophoresis and visualized under ultraviolet light.

**Table.1:** Primer sequences, product sizes, and annealing temperatures for β-lactamase-encoding genes in clinical isolates of *A. baumannii*

Target gene	Primer sequence (5′-3′)	Product size (bp)	Annealing Temperature (°C)	References
*bla*_TEM_	F: GAG TAT TCA ACA TTT CCG TG R: TAA TCA GTG AGG CAC CTA TC	848	47	[34]
*bla*_SHV_	F: TTA TCT CCC TGT TAG CCA CC R: GAT TTG CTG ATT TCG CTC GG	797	52	[35]
*bla*_CTX-M_	F: ACG CTG TTG TTA GGA AGT G R: TTG AGG CTG GGT GAA GT	759	52	[34]
*bla*_VEB_	F: CGA CTT CCA TTT CCC GAT GC R: GGA CTC TGC AAC AAA TAC GC	643	54	[36]
*bla*_PER_	F: GCA ACT GCT GCA ATA CTC GG R: ATG TGC GAC CAC AGT ACC AG	340	58	[37]
*bla*_GES_	F: TTG CAA TGT GCT CAA CGT TC R: TAT CAC AAC CAA TAT TGT CG	630	48	[38]
*bla*_IMP_	F: TTG ACA CTC CAT TTA CTG CTA R: TCA TTT GTT AAT TCA GAT GCA TA	172	50	[39]
*bla*_VIM_	F: GAG TTG CTT TTG ATT GAT ACA G R: TCG ATG AGA GTC CTT CTA GA	247	51	[39]
*bla*_NDM_	F: AAC ACA GCC TGA CTT TCG R: TGA TAT TGT CAC TGG TGT GG	111	53	[39]
IS*Aba1*	F: CAC GAA TGC AGA AGT TG R: CGA CGA ATA CTA TGA CAC	549	48	[25]
*bla*_OXA-51-like_	F: TAA TGC TTT GAT CGG CCT TG R: TGG ATT GCA CTT CAT CTT GG	353	53	[40]
*bla*_OXA−24−like_	F: GGT TAG TTG GCC CCC TTA AA R: AGT TGA GCG AAA AGG GGA T	246	54	[40]
*bla*_OXA-23-like_	F: GAT CGG ATT GGA GAA CCA GA R: ATT TCT GAC CGC ATT TCC AT	501	52	[40]
*bla*_OXA-58-like_	F: AAG TAT TGG GGC TTG TGC TG R: CCC CTC TGC GCT CTA CAT AC	599	56	[40]
IS*Aba1*/*bla*_OXA-23-like_	F: CAC GAA TGC AGA AGT TG R: ATT TCT GAC CGC ATT TCC AT	1404	50	[40]
IS*Aba1*/*bla*_OXA-51-like_	F: CAC GAA TGC AGA AGT TG R: TGG ATT GCA CTT CAT CTT GG	1223	52	[40]

### Random amplification of polymorphic DNA (RAPD)-PCR fingerprinting

The genetics relatedness of *bla*_OXA-24-like_-positive strains was assessed using RAPD-PCR analysis with a random oligonucleotide 5′-CCGCAGCCAA-3′ (TAG Copenhagen, Denmark). Amplification of random sequences was done by a conventional PCR as follows: initial denaturation at 94 °C for 10 min, followed by 30 cycles consisting of denaturation at 94 °C for 1 min, annealing at 43 °C for 1 min, extension at 72 °C for 3 min, and final extension at 72 °C for 10 min. The RAPD patterns was compared by GelCompar® II v.4.1 software (Applied Maths BVBA, Sint–Martens–Latem, Belgium) and clustered with unweighted pair group method (UPGMA).

### Statistical analysis

Data analysis was performed using the Minitab® statistical software package 17 (MINITAB, Inc., State College, Pennsylvania, USA). The *P* value ≤ 0.05 was considered statistically significant for the analysis.

## Results

### Clinical isolates and demographic characteristics of pediatric patients

A total of 60 non-replicate *A. baumannii* clinical isolates were collected from the Children’s Medical Center in a period of April 2019 to December 2020. 43 out of 60 (71.6%) and 17 out of 60 (28.3%) isolates of *A. baumannii* were belonged to male and female pediatric patients, respectively with an age range of 4 days to 14 years. The majority of isolates were obtained from patients admitted to the neonatal and pediatric intensive care units (NICU, PICU) (65%), followed by the other wards (35%). Distribution of collected isolates from the specimen sources was follows: CSF (6.66%), BAL (23.33%), tracheal tube (18.33%), blood (30%), urine (3.33%), central venous line (CVL) (3.33%), respiratory secretions (10%), drain discharge (3.33%), and dialysis fluid (1.7%). All isolates were molecularly confirmed as *A. baumannii* using both inherent *rpoB* and *bla*_OXA-51-like_ genes. Demographics and clinical features of pediatric patients are accessible in detail in a descriptive Additional file 1: Table S1.

### Antimicrobial susceptibility profile

Antimicrobial susceptibility testing was displayed that the highest resistance rate was for imipenem and meropenem (53, 88.3%), followed by cefotaxime, cefepime, ceftriaxone, levofloxacin (52, 86.67%), ceftazidime, amikacin, cotrimoxazole, ciprofloxacin (51, 85%), piperacillin-tazobactam (50, 83.3%), gentamicin (49, 81.67%), tetracycline (44, 73.3%), minocycline (34, 56.6%), doxycycline (32, 53.3%), ampicillin–sulbactam (29, 48.3%), and tigecycline (3, 5%). Fifty-two out of 60 isolates were resistant to both imipenem and meropenem, whereas 6 out of 60 isolates were sensitive to them. However, only one isolate was found to be sensitive to imipenem, and one was susceptible to meropenem.

All isolates were susceptible to colistin with MICs ranging from 0.125 to 2 µg/mL. The MIC_50_ and MIC_90_ of colistin were 0.5 and 2 µg/mL, respectively. Fifty-one out of 60 (83.3%) *A. baumannii* isolates were categorized as the MDR, and 24 out of 60 (40%) were extensively drug-resistant (XDR).

**Fig. 1:**
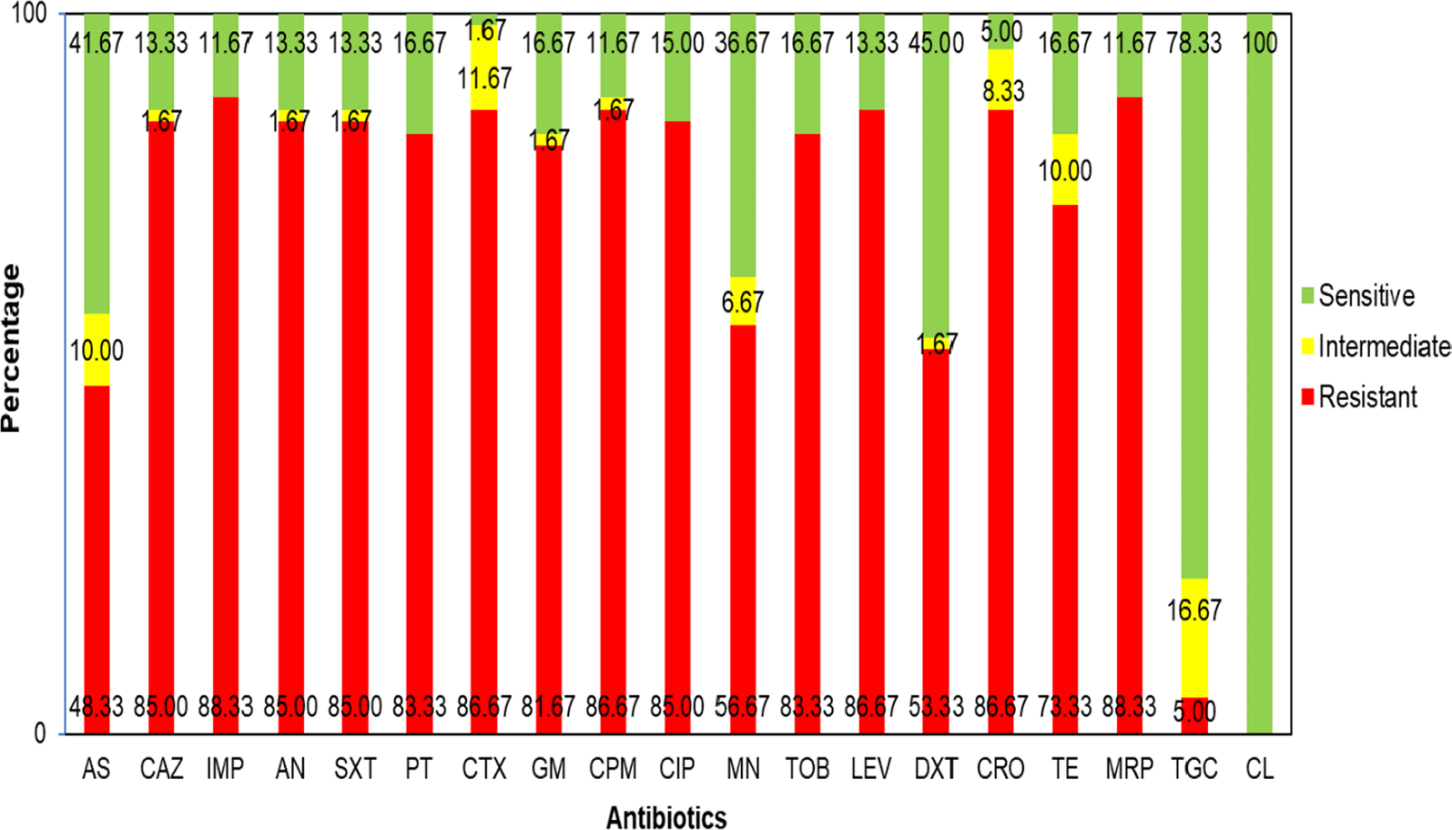
In vitro antimicrobial susceptibility profile of pediatric *A. baumannii* isolates. *AS* Ampicillin–Sulbactam, *CAZ* Ceftazidime, *IMP* Imipenem, *AN* Amikacin, *SXT* Trimethoprim–Sulfamethoxazole, *PT* Piperacillin–Tazobactam, *CTX* Cefotaxime, *GM* Gentamicin, *CPM* Cefepime, *CIP* Ciprofloxacin, *MN* Minocycline, *TOB* Tobramycin, *LEV* Levofloxacin, *DXT* Doxycycline, *CRO* Ceftriaxone, *TE* Tetracycline, *MRP* Meropenem, *TGC* Tigecycline, *CL* Colistin

Antimicrobial susceptibility testing for the isolates is summarized in Fig. 1.

### Characterization of β-lactamase resistance genes profile

**Fig. 2:**
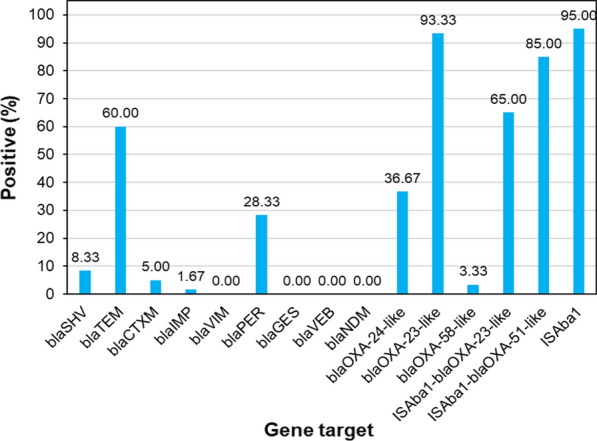
Distribution of β-lactamase-encoding genes and IS*Aba1* upstream of *bla*_OXA-23_ and *bla*_OXA-51_ in pediatric isolates of *A. baumannii*

Molecular analysis of β-lactamase resistance genes in *A. baumannii* isolates showed that 93.33% of the clinical isolates (n = 56) were positive for the *bla*_OXA-23-like_ gene, followed by *bla*_TEM_ 60% (n = 36), *bla*_OXA−24−like_ 36.67% (n = 22), *bla*_PER_ 28.33% (n = 17), *bla*_SHV_ 8.33% (n = 5), *bla*_CTX-M_ 5% (n = 3), *bla*_OXA-58-like_ 3.33% (n = 2), and *bla*_IMP_ 1.67% (n = 1). In contrast, we could not detect any *bla*_VIM_, *bla*_GES_, *bla*_VEB_, and *bla*_NDM_ genes. Although, the *bla*_OXA−51−like_ gene was identified in all *A. baumannii* isolates, we could not conclude the antibiotic resistance associated with its presence alone (*P* value > 0.05). The IS*Aba1* element was found in 95% of the isolates. However, IS*Aba1* was detected in the upstream of *bla*_OXA-23-like_ and *bla*_OXA-51-like_ genes in 65% and 85% of isolates, respectively. Distribution of β-lactamase-encoding genes and IS*Aba1* upstream of *bla*_OXA_ genes in pediatric isolates of *A. baumannii* is presented in Fig. 2 and Additional file 1: Table S1.

### RAPD genotyping of *A. baumannii*

**Fig. 3:**
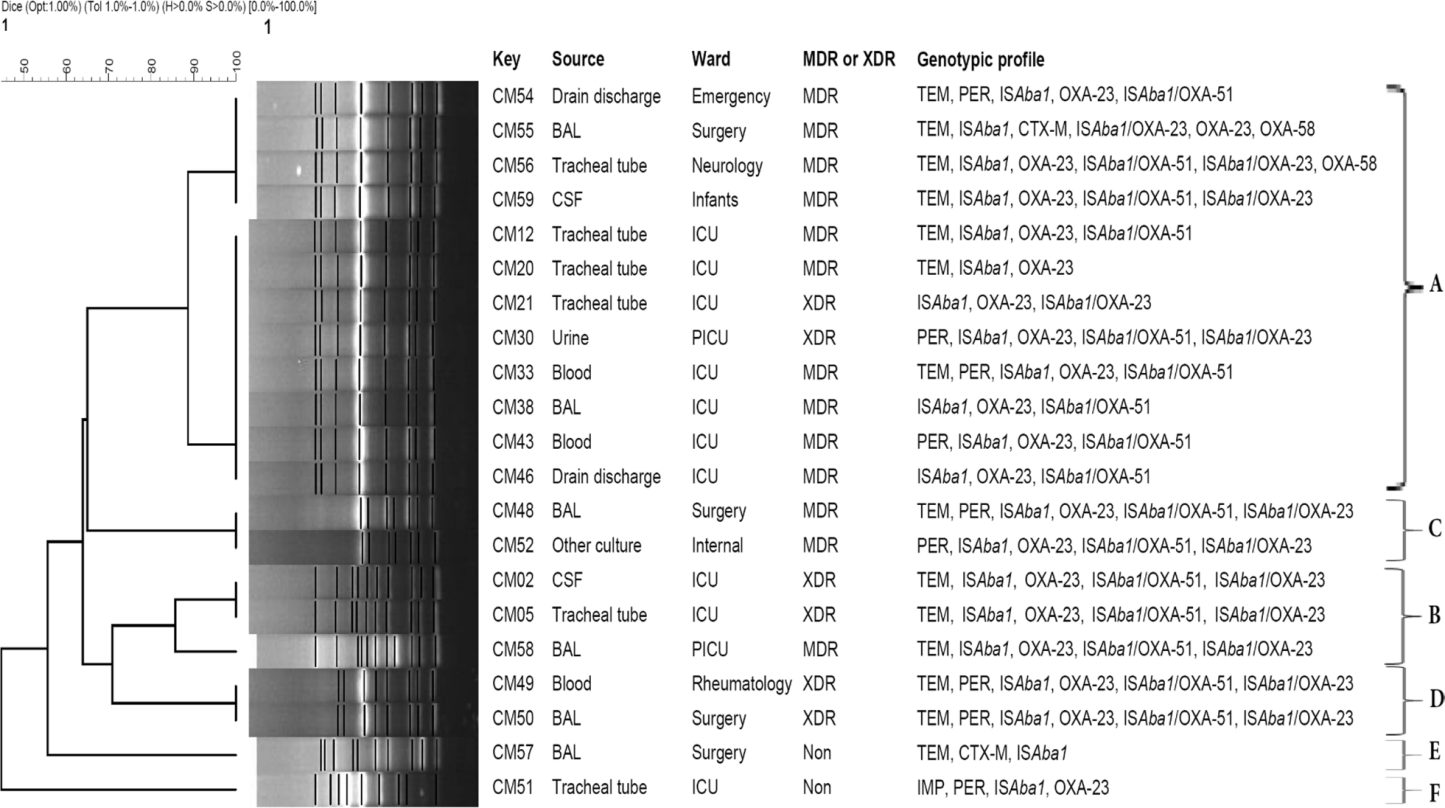
RAPD-PCR dendrogram of pediatric *A. baumannii* isolates identified 6 RAPD patterns, A to F

The *A. baumannii* isolates harboring *bla*_OXA-24-like_ were designated for further epidemiological analysis by RAPD-PCR. Dendrogram analysis by GelCompar software and UPGMA clustering method by the arithmetic mean calculation and the Dice coefficient (Dice similarity index) with an optimization of 0.5% and a tolerance of 1.5% were used for the similarity with a cut-off value of > 85% and considered the same RAPD type. According to the similarity index, we found 4 common-type (A–D) and 2 single-type (E and F). The major RAPD type A has included 12 strains, followed by RAPD type B with 3, RAPD type C with 2, and RAPD type D with 2 strains. Dendrogram of pediatric *A. baumannii* isolates based on RAPD-PCR analysis is shown in Fig. 3.

## Discussion

The incidence of MDR *A. baumannii* in pediatric patients is increasing worldwide as far as it has led to therapeutic challenges and has become an ongoing serious public health concern [13]. In this study, we described a high antibiotic resistance rate, rising MDR and XDR strains, and widespread prevalence of β-lactamase-encoding genes in *A. baumannii* isolated from pediatric patients.

Carbapenems, as the last-line group of β-lactam antibiotics, have been used to treat infections associated with MDR *A. baumannii* for a long time; however, carbapenem resistant *A. baumannii* (CRAB) has recently emerged and limited therapeutic options [14, 15]. We found that most isolates were resistant to carbapenem (imipenem and meropenem 88.3% for each, and both imipenem and meropenem 86.6%). A high rate of CRAB in pediatric has been explored in several similar studies [16, 17]. It has been reported that the drug resistance in CRAB is developed by several mechanisms, including production of hydrolyzing enzymes, horizontally transferring resistance determinants, changes in outer membrane permeability, and upregulation of the efflux systems. The major mechanism of carbapenem resistance in *A. baumannii* is related to the production of carbapenem-hydrolyzing β-lactamases, i.e., carbapenem-hydrolyzing class D β-lactamases (CHDLs), class B metallo-β-lactamases (MBLs), and class A carbapenemases [18]. Distribution of the CHDLs, MBLs, and ESBLs genes has been reported with different prevalence in recent published papers [19–21]. We could not find *A. baumannii* isolates harboring *bla*_VIM_, *bla*_GES_, *bla*_VEB_, and *bla*_NDM_ genes. In contrast, a high prevalence rate of *bla*_NDM_ gene has been observed in CRAB isolates from Egyptian patients [22]. The *bla*_OXA-23-like_ was more prevalent in our study, which is in agreement with previous studies [17, 23]. Moreover, we also detected the *bla*_OXA-24-like_ and *bla*_OXA-58-like_ genes, but in lower frequencies, corresponding with similar reports [20, 24]. Although the catalytic activity of OXA-type carbapenemases is far less than MBLs, their expression can be influenced by upstream existence of insertion sequences (ISs) elements such as IS*Aba1* resulting in increased resistance to carbapenems [25]. Most isolates carried IS*Aba1*; however, 85% of isolates harbored IS*Aba1*-*bla*_OXA−51−like_ and 65% were also positive for IS*Aba1*/*bla*_OXA-23-like_. We found a statistically significant relationship between the presence of IS*Aba1* upstream of *bla*_OXA-23-like_ and *bla*_OXA-51-like_ and carbapenem resistance (*P* value < 0.05). Over 90% of isolates (54 out of 60) were resistant to carbapenems (imipenem and meropenem) with the presence of IS*Aba1* upstream of *bla*_OXA-23-like_ and *bla*_OXA-51-like_; though, four isolates despite the existence of IS*Aba1* upstream of *bla*_OXA-23-like_ and *bla*_OXA-51-like_ were susceptible to carbapenems; while it might be expected that the co-existence of the determinants should confer resistance to the carbapenems, as described by Turton et al. [25]. Another study has suggested that IS*Aba1*-bla_OXA-51-like_ alone is insufficient to confer resistance to carbapenems [26]. Nevertheless, it has not been completely clarified that the cause of the contradiction in carbapenem susceptibility levels in isolates harboring the IS*Aba1*-*bla*_OXA-51-like_ gene [27].

Numerous reports have indicated that colistin (polymyxin E) and tigecycline, the only active antibiotics against *A. baumannii*, have become the last resort of treatment for MDR *A. baumannii *[28]. Now, resistance to colistin is relatively low; however, colistin-resistant strains have been introduced from different regions of the world [29]. A recent study has reported inhibitory potential effects of methylene blue for improving colistin susceptibility in the treatment of colistin-resistant strains of *A. baumannii *[30]. We investigated the in vitro activity of colistin, indicating that all *A. baumannii* isolates were susceptible to colistin according to the MIC50 and MIC90 values of < 2 µg/ml. Significantly, both MDR and XDR *A. baumannii* were susceptible to colistin which are in concordance with previous studies [13, 31].

RAPD is a simple and rapid technique and possesses high discriminative power for source tracking of bacteria, and it has been used in epidemiological studies of *Acinetobacter* species to identify circulating clones in clinical settings [32, 33]. The 21 *bla*_OXA-24-like_-positive isolates were distributed into 4 RAPD types belonging to four clusters (A, B, C, D) and two additional RAPD types E and F. Twelve strains were categorized in cluster A. All strains belonging to cluster A were MDR. All strains of cluster A were positive for IS*Aba1* and *bla*_OXA-23-like_, and the genetic profile of the strains was also varied. However, antibiotic resistance profiles were different between the isolates within the same group. Several strains with the same genetic profile and common origin were observed in clusters B and D. All strains of clusters E and F were susceptible to all antibiotic groups. Significantly, strains were collected from different infection sites were genetically categorized in the same cluster, suggesting the same origin and upsurging threat of multiple infection abilities for these genotypes. We successfully applied RAPD for distinguishing the genetic diversity of *A. baumannii* clones circulating in clinical setting.

Epidemiological studies on the dissemination of drug-resistant clones and their resistance gene profile are prerequisites to control infection and prevent policies in clinical settings.

There were a number of limitations to the current study; (a) the present literature was limited to single-center experiences, (b) our population size was small, (c) there was not enough data about pediatric patients’ records. Further researches are required to detect the expression level of resistance genes by real-time PCR assay. Other methods such as next-generation sequencing (NGS) can also be considered for well understanding the molecular mechanisms of resistance.

## Conclusions

The distribution of resistance determinants in *A. baumannii* isolates is a great concern for our pediatric center and would become therapeutic challenges soon.

The high prevalence of the MDR clones circulating in different wards is a problematic issue for infection control, surveillance programs, and health care strategies, especially in the pediatric population. It is recommended that the development of novel therapeutic strategies or reassessment of old drugs is scheduled to make insight for pediatricians to address and find alternative antibiotics with high efficiency. In addition, longitudinal studies are needed for continuous monitoring and tracking dispersion dynamics of successful clones associated with the persistence of drug-resistant phenotypes.

## Supplementary Information

**Additional file 1: Table S1.** Clinical and demographics data of pediatricpatients, drug resistance profile, and distribution pattern of β-lactamase genes in *A. baumannii* isolates.

## Data Availability

All data generated or analyzed during this study were included in this article.

## Abbreviations

*A. baumannii*: *Acinetobacter baumannii*
RAPD: Random amplification of polymorphic DNA
MDR: Multidrug-resistant
XDR: Extensively drug-resistant
*bla*: Beta-lactamase
OXA: Oxacillinase
IS: Insertion sequence
ESBLs: Extended spectrum beta-lactamases
TEM: Temoniera, patient’s name
SHV: Sulfhydryl variable
CTX-M: Referred to its preferential hydrolytic activity against cefotaxime (CTX as its acronym, -M from Munich)
PER: Pseudomonas extended resistant
VEB: Vietnam extended-spectrum β-lactamase
GES: Guiana extended-spectrum β-lactamase
IMP: Imipenemase
VIM: Verona integron-encoded metallo-β-lactamase
GIM: German imipenemase
NDM: New Delhi metallo-β-lactamase
AmpC: Ampicillinase C
*rpoB*: RNA polymerase β subunit
CLSI: Clinical and Laboratory Standards Institute
MIC: Minimum inhibitory concentration
ATCC: American Type Culture Collection
UPGMA: Unweighted pair group method
ICU: Intensive care unit
NICU: neonatal intensive care unit
PICU: Pediatric intensive care unit
OPD: Outpatient department
CSF: Cerebrospinal fluid
BAL: Bronchoalveolar lavage
CVL: Central venous line
CRAM: Carbapenem resistant *A. baumannii*
CHDLs: Carbapenem-hydrolyzing class D β-lactamases
