# Supplementary material for: Prevalence of β-lactamase-encoding genes and molecular typing of Acinetobacter baumannii isolates carrying carbapenemase OXA-24 in children
Source: Ann Clin Microbiol Antimicrob. 2021 Oct 26;20:75. doi: 10.1186/s12941-021-00480-5 (PMC8549256; doi:10.1186/s12941-021-00480-5)
Supplement: Supplementary file 1 — Additional file 1: Table S1. Clinical and demographics data of pediatricpatients, drug resistance profile, and distribution pattern of β-lactamase genes in A. baumannii isolates. [file 12941_2021_480_MOESM1_ESM.docx]

| Table S1. Clinical and demographics data of pediatric patients, drug resistance profile, and distribution pattern of β-lactamase genes in *A. baumannii* isolates | P6 | 2 years | Female | PICU | Blood | CAZ, IMP, AN, SXT, PT, CTX, GM, CPM, CIP, MN, TOB, LEV, DXT, CRO, MRP | Yes | No | No | Yes | No | No | No | No | No | No | No | No | Yes | No | No | Yes | Yes |
| --- | --- | --- | --- | --- | --- | --- | --- | --- | --- | --- | --- | --- | --- | --- | --- | --- | --- | --- | --- | --- | --- | --- | --- |
|  | P5 | 8 days | Male | ICU | Tracheal tube | AS, CAZ, IMP, AN, SXT, PT, CTX, GM, CPM, CIP, MN, TOB, LEV, DXT, CRO, TE, MRP | Yes | Yes | No | Yes | No | No | No | No | No | No | No | Yes | Yes | No | Yes | Yes | Yes |
|  | P4 | 2 months | Male | Rheumatology | BAL | AS, CAZ, IMP, SXT, PT, CTX, GM, CPM, CIP, MN, TOB, LEV, DXT, CRO, MRP | Yes | No | No | No | No | No | No | No | No | No | No | No | Yes | No | Yes | Yes | Yes |
|  | P3 | 1 year | Female | PICU | BAL | AS, CAZ, IMP, AN, SXT, PT, CTX, GM, CPM, CIP, MN, TOB, LEV, DXT, CRO, TE, MRP | Yes | Yes | No | Yes | No | No | No | No | No | No | No | No | Yes | No | No | Yes | Yes |
|  | P2 | 4 days | Female | ICU | CSF | AS, CAZ, IMP, AN, SXT, PT, CTX, GM, CPM, CIP, MN, TOB, LEV, DXT, CRO, TE, MRP | Yes | Yes | No | Yes | No | No | No | No | No | No | No | Yes | Yes | No | Yes | Yes | Yes |
|  | P1 | 2 months | Male | Rheumatology | IV catheter | CAZ, IMP, AN, SXT, PT, CTX, GM, CPM, CIP, MN, TOB, LEV, DXT, CRO, MRP | Yes | No | No | No | No | No | No | No | No | No | No | No | Yes | No | No | Yes | Yes |
|  | **Patient** | **Age** | **Gender** | **Ward** | **Source** | **Resistance pattern** | **MDR** | **XDR** | ***bla*_SHV_** | ***bla*_TEM_** | ***bla*_CTX-M_** | ***bla*_IMP_** | ***bla*_VIM_** | ***bla*_PER_** | ***bla*_GES_** | ***bla*_VEB_** | ***bla*_NDM_** | ***bla*_OXA-24-like_** | ***bla*_OXA-23-like_** | ***bla*_OXA-58-like_** | **IS*Aba1*-*bla*_OXA-23-like_** | **IS*Aba1*-*bla*_OXA-51-like_** | **IS*Aba1*** |
| Table S1. Cont. | P12 | 6 years | Male | PICU | Tracheal tube | AS, CAZ, IMP, AN, SXT, CTX, CPM, CIP, TOB, LEV, CRO, TE, MRP | Yes | No | No | Yes | No | No | No | No | No | No | No | Yes | Yes | No | No | Yes | Yes |
|  | P11 | 16 months | Male | PICU | BAL | AS, CAZ, IMP, SXT, PT, CTX, CPM, CIP, MN, TOB, LEV, DXT, CRO, TE, MRP | Yes | No | No | Yes | No | No | No | No | No | No | No | No | Yes | No | Yes | Yes | Yes |
|  | P10 | 6 months | Male | Surgery | Esophagectomy | AS, CAZ, IMP, AN, SXT, PT, CTX, GM, CPM, CIP, MN, TOB, LEV, DXT, CRO, TE, MRP | Yes | Yes | No | Yes | No | No | No | No | No | No | No | No | Yes | No | Yes | Yes | Yes |
|  | P9 | 5 days | Male | ICU | Blood | AS, CAZ, IMP, AN, SXT, PT, CTX, GM, CPM, CIP, MN, TOB, LEV, DXT, CRO, TE, MRP | Yes | Yes | No | Yes | No | No | No | No | No | No | No | No | Yes | No | Yes | Yes | Yes |
|  | P8 | 6 months | Male | Rheumatology | Urine | CAZ, IMP, AN, SXT, PT, CTX, GM, CPM, CIP, MN, TOB, LEV, DXT, CRO, TE, MRP | Yes | No | No | No | No | No | No | No | No | No | No | No | Yes | No | Yes | Yes | Yes |
|  | P7 | 1 year | Male | PICU | Blood | MN, TOB, LEV, DXT, TE, MRP | No | No | No | No | No | No | No | No | No | No | No | No | Yes | No | Yes | Yes | Yes |
|  | **Patient** | **Age** | **Gender** | **Ward** | **Source** | **Resistance pattern** | **MDR** | **XDR** | ***bla*_SHV_** | ***bla*_TEM_** | ***bla*_CTX-M_** | ***bla*_IMP_** | ***bla*_VIM_** | ***bla*_PER_** | ***bla*_GES_** | ***bla*_VEB_** | ***bla*_NDM_** | ***bla*_OXA-24-like_** | ***bla*_OXA-23-like_** | ***bla*_OXA-58-like_** | **IS*Aba1*-*bla*_OXA-23-like_** | **IS*Aba1*-*bla*_OXA-51-like_** | **IS*Aba1*** |
| Table S1. Cont. | P18 | 2 years | Male | PICU | Central venous line | CAZ, IMP, AN, SXT, PT, CTX, GM, CPM, CIP, MN, TOB, LEV, DXT, CRO, TE, MRP | Yes | No | Yes | Yes | No | No | No | No | No | No | No | No | Yes | No | Yes | Yes | Yes |
|  | P17 | 1 year | Female | ICU | Blood | CAZ, IMP, AN, SXT, PT, CTX, GM, CPM, CIP, MN, TOB, LEV, DXT, CRO, TE, MRP | Yes | Yes | Yes | Yes | No | No | No | No | No | No | No | No | Yes | No | Yes | No | Yes |
|  | P16 | 24 days | Male | ICU | Blood | AS, CAZ, IMP, AN, SXT, PT, CTX, GM, CPM, CIP, MN, TOB, LEV, DXT, CRO, TE, MRP | Yes | Yes | No | Yes | No | No | No | No | No | No | No | No | Yes | No | Yes | Yes | Yes |
|  | P15 | 3 years | Male | PICU | Blood | AS, CAZ, IMP, AN, SXT, PT, CTX, GM, CPM, CIP, MN, TOB, LEV, DXT, CRO, TE, MRP | Yes | Yes | No | No | No | No | No | No | No | No | No | No | Yes | No | No | Yes | Yes |
|  | P14 | 14 days | Male | ICU | Tracheal tube | CAZ, IMP, AN, SXT, PT, CTX, GM, CPM, CIP, TOB, LEV, CRO, TE, MRP | Yes | No | No | Yes | No | No | No | No | No | No | No | No | Yes | No | No | Yes | Yes |
|  | P13 | 11 years | Male | Surgery | CSF | AS, CAZ, IMP, AN, SXT, PT, CTX, GM, CPM, CIP, MN, TOB, LEV, DXT, CRO, TE, MRP | Yes | Yes | No | Yes | No | No | No | No | No | No | No | No | Yes | No | Yes | Yes | Yes |
|  | **Patient** | **Age** | **Gender** | **Ward** | **Source** | **Resistance pattern** | **MDR** | **XDR** | ***bla*_SHV_** | ***bla*_TEM_** | ***bla*_CTX-M_** | ***bla*_IMP_** | ***bla*_VIM_** | ***bla*_PER_** | ***bla*_GES_** | ***bla*_VEB_** | ***bla*_NDM_** | ***bla*_OXA-24-like_** | ***bla*_OXA-23-like_** | ***bla*_OXA-58-like_** | **IS*Aba1*-*bla*_OXA-23-like_** | **IS*Aba1*-*bla*_OXA-51-like_** | **IS*Aba1*** |
| Table S1. Cont. | P24 | 4 months | Male | Rheumatology | Blood | CAZ, IMP, AN, SXT, PT, CTX, GM, CPM, CIP, MN, TOB, LEV, DXT, CRO, TE, MRP | Yes | No | No | Yes | No | No | No | No | No | No | No | No | Yes | No | Yes | Yes | Yes |
|  | P23 | 7 years | Female | PICU | BAL | CAZ, IMP, AN, SXT, PT, CTX, GM, CPM, CIP, MN, TOB, LEV, DXT, CRO, TE, MRP | Yes | Yes | No | Yes | No | No | No | No | No | No | No | No | Yes | No | Yes | Yes | Yes |
|  | P22 | 4 years | Male | PICU | BAL | CAZ, IMP, AN, SXT, PT, CTX, GM, CPM, CIP, MN, TOB, LEV, DXT, CRO, TE, MRP | Yes | Yes | No | Yes | No | No | No | No | No | No | No | No | Yes | No | Yes | Yes | Yes |
|  | P21 | 20 days | Male | ICU | Tracheal tube | CAZ, IMP, AN, SXT, PT, CTX, GM, CPM, CIP, TOB, LEV, DXT, CRO, TE, MRP | Yes | Yes | No | No | No | No | No | No | No | No | No | Yes | Yes | No | Yes | No | Yes |
|  | P20 | 12 days | Male | ICU | Tracheal tube | AS, CAZ, IMP, AN, SXT, PT, CTX, GM, CPM, CIP, TOB, LEV, CRO, TE, MRP | Yes | No | No | Yes | No | No | No | No | No | No | No | Yes | Yes | No | No | No | Yes |
|  | P19 | 4 months | Male | ICU | Tracheal tube | AS, CAZ, IMP, AN, SXT, PT, CTX, GM, CPM, CIP, MN, TOB, LEV, DXT, CRO, TE, MRP | Yes | Yes | No | No | No | No | No | No | No | No | No | No | Yes | No | Yes | Yes | Yes |
|  | **Patient** | **Age** | **Gender** | **Ward** | **Source** | **Resistance pattern** | **MDR** | **XDR** | ***bla*_SHV_** | ***bla*_TEM_** | ***bla*_CTX-M_** | ***bla*_IMP_** | ***bla*_VIM_** | ***bla*_PER_** | ***bla*_GES_** | ***bla*_VEB_** | ***bla*_NDM_** | ***bla*_OXA-24-like_** | ***bla*_OXA-23-like_** | ***bla*_OXA-58-like_** | **IS*Aba1*-*bla*_OXA-23-like_** | **IS*Aba1*-*bla*_OXA-51-like_** | **IS*Aba1*** |
| Table S1. Cont. | P30 | 1 months | Male | Infants | Urine | AS, CAZ, IMP, AN, SXT, PT, CTX, GM, CPM, CIP, TOB, LEV, CRO, MRP | Yes | Yes | No | No | No | No | No | Yes | No | No | No | Yes | Yes | No | Yes | Yes | Yes |
|  | P29 | 8 years | Male | PICU | Blood | AS, CAZ, IMP, AN, SXT, PT, CTX, GM, CPM, CIP, MN, TOB, LEV, DXT, CRO, TE, MRP | Yes | No | No | Yes | No | No | No | Yes | No | No | No | No | Yes | No | Yes | Yes | Yes |
|  | P28 | 3 years | Female | PICU | BAL | AS, CAZ, IMP, AN, SXT, PT, CTX, GM, CPM, CIP, MN, TOB, LEV, DXT, CRO, TE, MRP | Yes | Yes | Yes | No | No | No | No | No | No | No | No | No | Yes | No | Yes | Yes | Yes |
|  | P27 | 11 months | Female | PICU | BAL | CAZ, IMP, AN, SXT, PT, CTX, GM, CPM, CIP, MN, TOB, LEV, DXT, CRO, TE, MRP | Yes | Yes | Yes | No | No | No | No | Yes | No | No | No | No | Yes | No | Yes | Yes | Yes |
|  | P26 | 4 years | Male | PICU | BAL | CAZ, IMP, AN, SXT, PT, CTX, GM, CPM, CIP, MN, TOB, LEV, DXT, CRO, TE, MRP | Yes | Yes | Yes | No | Yes | No | No | No | No | No | No | No | Yes | No | Yes | Yes | Yes |
|  | P25 | 20 months | Male | ICU | Blood | CAZ, IMP, AN, SXT, PT, CTX, GM, CPM, CIP, MN, TOB, LEV, CRO, TE, MRP | Yes | No | No | No | No | No | No | No | No | No | No | Yes | No | No | No | No | No |
|  | **Patient** | **Age** | **Gender** | **Ward** | **Source** | **Resistance pattern** | **MDR** | **XDR** | ***bla*_SHV_** | ***bla*_TEM_** | ***bla*_CTX-M_** | ***bla*_IMP_** | ***bla*_VIM_** | ***bla*_PER_** | ***bla*_GES_** | ***bla*_VEB_** | ***bla*_NDM_** | ***bla*_OXA-24-like_** | ***bla*_OXA-23-like_** | ***bla*_OXA-58-like_** | **IS*Aba1*-*bla*_OXA-23-like_** | **IS*Aba1*-*bla*_OXA-51-like_** | **IS*Aba1*** |
| Table S1. Cont. | P36 | 3 months | Male | GI | Blood | TE | No | No | No | No | No | No | No | Yes | No | No | No | No | Yes | No | No | No | Yes |
|  | P35 | 2 years | Male | ICU | CSF | IMP, AN, CRO, MRP | No | No | No | No | No | No | No | Yes | No | No | No | No | Yes | No | Yes | Yes | Yes |
|  | P34 | 10 years | Male | Emergency | Sputum | CAZ, IMP, AN, SXT, PT, CTX, GM, CPM, CIP, MN, TOB, LEV, CRO, TE, MRP | Yes | Yes | No | No | No | No | No | Yes | No | No | No | No | Yes | No | No | Yes | Yes |
|  | P33 | 5 years | Male | ICU | Blood | AS, CAZ, IMP, AN, SXT, PT, CTX, GM, CPM, CIP, TOB, LEV, CRO, TE, MRP | Yes | No | No | Yes | No | No | No | Yes | No | No | No | Yes | Yes | No | No | Yes | Yes |
|  | P32 | 11 years | Female | ICU | Throat | AS, CAZ, IMP, AN, SXT, PT, CTX, GM, CPM, CIP, MN, LEV, CRO, TE, MRP | Yes | No | No | No | No | No | No | Yes | No | No | No | No | Yes | No | Yes | Yes | Yes |
|  | P31 | 12 years | Male | ICU | Blood | CAZ, IMP, AN, SXT, PT, CTX, CPM, CIP, MN, LEV, DXT, CRO, TE, MRP | Yes | No | No | No | No | No | No | Yes | No | No | No | No | No | No | Yes | Yes | Yes |
|  | **Patient** | **Age** | **Gender** | **Ward** | **Source** | **Resistance pattern** | **MDR** | **XDR** | ***bla*_SHV_** | ***bla*_TEM_** | ***bla*_CT-XM_** | ***bla*_IMP_** | ***bla*_VIM_** | ***bla*_PER_** | ***bla*_GES_** | ***bla*_VEB_** | ***bla*_NDM_** | ***bla*_OXA-24-like_** | ***bla*_OXA-23-like_** | ***bla*_OXA-58-like_** | **IS*Aba1*-*bla*_OXA-23-like_** | **IS*Aba1*-*bla*_OXA-51-like_** | **IS*Aba1*** |
| Table S1. Cont. | P42 | 2 months | Female | ICU | Blood | - | No | No | No | Yes | No | No | No | No | No | No | No | No | Yes | No | Yes | Yes | No |
|  | P41 | 9 days | Male | ICU | Tracheal tube | CAZ, IMP, AN, SXT, PT, CTX, GM, CPM, CIP, MN, TOB, LEV, DXT, CRO, TE, MRP, TGC | Yes | No | No | Yes | No | No | No | No | No | No | No | No | Yes | No | Yes | Yes | Yes |
|  | P40 | 8 days | Female | ICU | Tracheal tube | AS, CAZ, IMP, AN, SXT, PT, CTX, GM, CPM, CIP, MN, TOB, LEV, DXT, CRO, TE, MRP, TGC | Yes | Yes | No | Yes | No | No | No | No | No | No | No | No | Yes | No | Yes | Yes | Yes |
|  | P39 | 10 months | Male | ICU | Nasopharyngeal secretions | TOB | No | No | No | No | No | No | No | No | No | No | No | No | Yes | No | Yes | Yes | Yes |
|  | P38 | 1 years | Male | ICU | BAL | AS, CAZ, IMP, AN, SXT, PT, CTX, GM, CPM, CIP, TOB, LEV, CRO, TE, MRP | Yes | No | No | No | No | No | No | No | No | No | No | Yes | Yes | No | No | Yes | Yes |
|  | P37 | 1 month | Female | ICU | Blood | AS, CAZ, IMP, AN, SXT, PT, CTX, GM, CPM, CIP, MN, TOB, LEV, DXT, CRO, TE, MRP | Yes | Yes | No | No | No | No | No | Yes | No | No | No | No | Yes | No | Yes | Yes | Yes |
|  | **Patient** | **Age** | **Gender** | **Ward** | **Source** | **Resistance pattern** | **MDR** | **XDR** | ***bla*_SHV_** | ***bla*_TEM_** | ***bla*_CTX-M_** | ***bla*_IMP_** | ***bla*_VIM_** | ***bla*_PER_** | ***bla*_GES_** | ***bla*_VEB_** | ***bla*_NDM_** | ***bla*_OXA-24-like_** | ***bla*_OXA-23-like_** | ***bla*_OXA-58-like_** | **IS*Aba1*-*bla*_OXA-23-like_** | **IS*Aba1*-*bla*_OXA-51-like_** | **IS*Aba1*** |
| Table S1. Cont. | P48 | 11 months | Male | Surgery | BAL | AS, CAZ, IMP, AN, SXT, PT, CTX, GM, CPM, CIP, TOB, LEV, CRO, TE, MRP | Yes | No | No | Yes | No | No | No | Yes | No | No | No | Yes | Yes | No | Yes | Yes | Yes |
|  | P47 | 4 months | Male | ICU | Dialysis fluid | AS, CAZ, IMP, AN, SXT, PT, CTX, GM, CPM, CIP, MN, TOB, LEV, DXT, CRO, TE, MRP | Yes | Yes | No | Yes | No | No | No | No | No | No | No | No | Yes | No | No | Yes | Yes |
|  | P46 | 29 days | Female | ICU | Drain discharge | AS, CAZ, IMP, AN, SXT, PT, CTX, GM, CPM, CIP, TOB, LEV, CRO, MRP | Yes | No | No | No | No | No | No | No | No | No | No | Yes | Yes | No | No | Yes | Yes |
|  | P45 | 9 days | Male | ICU | Blood | CAZ, IMP, AN, SXT, PT, CTX, GM, CPM, CIP, MN, TOB, LEV, DXT, CRO, TE, MRP | Yes | Yes | No | Yes | No | No | No | No | No | No | No | No | Yes | No | No | Yes | Yes |
|  | P44 | 4 months | Male | OPD | Throat | AN | No | No | No | Yes | No | No | No | No | No | No | No | No | Yes | No | No | Yes | Yes |
|  | P43 | 4 days | Male | ICU | Blood | AS, CAZ, IMP, AN, SXT, PT, CTX, GM, CPM, CIP, TOB, LEV, CRO, MRP | Yes | No | No | No | No | No | No | Yes | No | No | No | Yes | Yes | No | No | Yes | Yes |
|  | **Patient** | **Age** | **Gender** | **Ward** | **Source** | **Resistance pattern** | **MDR** | **XDR** | ***bla*_SHV_** | ***bla*_TEM_** | ***bla*_CTX-M_** | ***bla*_IMP_** | ***bla*_VIM_** | ***bla*_PER_** | ***bla*_GES_** | ***bla*_VEB_** | ***bla*_NDM_** | ***bla*_OXA-24-like_** | ***bla*_OXA-23-like_** | ***bla*_OXA-58-like_** | **IS*Aba1*-*bla*_OXA-23-like_** | **IS*Aba1*-*bla*_OXA-51-like_** | **IS*Aba1*** |
| Table S1. Cont. | P54 | 3 months | Male | Emergency | Drain discharge | AS, CAZ, IMP, AN, SXT, PT, CTX, GM, CPM, CIP, TOB, LEV, CRO, MRP | Yes | No | No | Yes | No | No | No | Yes | No | No | No | Yes | Yes | No | No | Yes | Yes |
|  | P53 | 5 months | Male | Emergency | Throat | - | No | No | No | Yes | No | No | No | No | No | No | No | No | Yes | No | Yes | Yes | No |
|  | P52 | 2 years | Female | Internal | Blood | CAZ, IMP, AN, SXT, PT, CTX, GM, CPM, CIP, TOB, LEV, CRO, TE, MRP | Yes | No | No | No | No | No | No | Yes | No | No | No | Yes | No | No | Yes | Yes | Yes |
|  | P51 | 5 days | Male | ICU | Tracheal tube | CAZ, IMP, AN, SXT, CTX, CPM, CRO, MRP | No | No | No | No | No | Yes | No | Yes | No | No | No | Yes | Yes | No | No | No | Yes |
|  | P50 | 1 month | Male | Surgery | BAL | AS, CAZ, IMP, AN, SXT, PT, CTX, GM, CPM, CIP, TOB, LEV, DXT, CRO, TE, MRP | Yes | Yes | No | Yes | No | No | No | Yes | No | No | No | Yes | Yes | No | Yes | Yes | Yes |
|  | P49 | 3 months | Female | Rheumatology | Blood | AS, CAZ, IMP, AN, SXT, PT, CTX, GM, CPM, CIP, TOB, LEV, CRO, TE, MRP | Yes | Yes | No | Yes | No | No | No | Yes | No | No | No | Yes | Yes | No | Yes | Yes | Yes |
|  | **Patient** | **Age** | **Gender** | **Ward** | **Source** | **Resistance pattern** | **MDR** | **XDR** | ***bla*_SHV_** | ***bla*_TEM_** | ***bla*_CTX-M_** | ***bla*_IMP_** | ***bla*_VIM_** | ***bla*_PER_** | ***bla*_GES_** | ***bla*_VEB_** | ***bla*_NDM_** | ***bla*_OXA-24-like_** | ***bla*_OXA-23-like_** | ***bla*_OXA-58-like_** | **IS*Aba1*-*bla*_OXA-23-like_** | **IS*Aba1*-*bla*_OXA-51-like_** | **IS*Aba1*** |
| Table S1. Cont. | P60 | 1 year | Female | PICU | Tracheal tube | AS, CAZ, IMP, AN, SXT, PT, CTX, GM, CPM, CIP, MN, TOB, LEV, CRO, TE | Yes | No | No | Yes | No | No | No | No | No | No | No | No | Yes | No | No | No | Yes |
|  | P59 | 16 days | Male | Infants | CSF | CAZ, IMP, AN, SXT, PT, CTX, GM, CPM, CIP, TOB, LEV, CRO, TE, MRP | Yes | No | No | Yes | No | No | No | No | No | No | No | Yes | Yes | No | Yes | Yes | Yes |
|  | P58 | 8 years | Female | PICU | BAL | IMP, PT, CTX, GM, CPM, CIP, LEV, TE, MRP | Yes | No | No | Yes | No | No | No | No | No | No | No | Yes | Yes | No | Yes | Yes | Yes |
|  | P57 | 10 years | Female | Surgery | BAL | GM | No | No | No | Yes | Yes | No | No | No | No | No | No | Yes | No | No | No | No | Yes |
|  | P56 | 6 years | Male | Neurology | Tracheal tube | AS, CAZ, IMP, AN, SXT, PT, CTX, GM, CPM, CIP, TOB, LEV, CRO, MRP | Yes | No | No | Yes | No | No | No | No | No | No | No | Yes | Yes | Yes | Yes | Yes | Yes |
|  | P55 | 14 years | Male | Surgery | BAL | AS, CAZ, IMP, AN, SXT, PT, CTX, GM, CPM, CIP, TOB, LEV, CRO, MRP | Yes | No | No | Yes | Yes | No | No | No | No | No | No | Yes | Yes | Yes | Yes | No | Yes |
|  | **Patient** | **Age** | **Gender** | **Ward** | **Source** | **Resistance pattern** | **MDR** | **XDR** | ***bla*_SHV_** | ***bla*_TEM_** | ***bla*_CTX-M_** | ***bla*_IMP_** | ***bla*_VIM_** | ***bla*_PER_** | ***bla*_GES_** | ***bla*_VEB_** | ***bla*_NDM_** | ***bla*_OXA-24-like_** | ***bla*_OXA-23-like_** | ***bla*_OXA-58-like_** | **IS*Aba1*-*bla*_OXA-23-like_** | **IS*Aba1*-*bla*_OXA-51-like_** | **IS*Aba1*** |
